# Cosmetic Bont‐A–Induced Transient Esotropia: A Case Expanding the Clinical Spectrum of Lateral Rectus Involvement

**DOI:** 10.1111/jocd.71093

**Published:** 2026-07-22

**Authors:** Tunahan Akyol, Emine Seker Un, Osman Parca

**Affiliations:** ^1^ Department of Ophthalmology Buldan State Hospital Denizli Türkiye; ^2^ Department of Ophthalmology Göz Akademi Hospital Denizli Türkiye; ^3^ Department of Ophthalmology Pamukkale University Faculty of Medicine Denizli Türkiye

**Keywords:** botulinum toxin, case report, cosmetic injection, diplopia, esotropia, lateral rectus weakness, ocular misalignment, periocular BoNT‐A complication, toxin diffusion, transient strabismus

## Abstract

**Purpose:**

To present a rare case of transient esodeviation and binocular diplopia caused by unintended medial rectus involvement following periocular botulinum toxin type A (BoNT‐A) injection performed in a non‐medical setting.

**Case Report:**

A 39‐year‐old female with no systemic disease presented with a 1‐day history of painless horizontal binocular diplopia. Five days before symptom onset, she had received a cosmetic BoNT‐A injection into the glabellar, forehead, and lateral canthal regions at a non‐medical beauty center. Ophthalmic examination revealed a visual acuity of 0.0 logMAR bilaterally, normal anterior and posterior segment findings, and a 10‐prism‐diopter esodeviation in primary gaze. Extraocular motility demonstrated full ductions without restrictive patterns. Worth four‐dot testing showed right‐eye suppression, and stereopsis was markedly reduced. Cranial nerve and neurological examinations were normal. Orbital–cranial CT and MRI were unremarkable. The clinical picture was consistent with unintended diffusion of the toxin, leading to transient medial rectus overaction. The patient was managed conservatively with prism correction. Prism strength was gradually reduced over follow‐up (8 PD → 6 PD → 2 PD), and prisms were discontinued by month four. Full resolution of diplopia occurred at month six, consistent with the expected timeframe of neuromuscular recovery after BoNT‐A exposure.

**Conclusion:**

Medial rectus involvement following cosmetic BoNT‐A injection is extremely rare but may result in symptomatic diplopia and functional visual impairment. This case emphasizes that botulinum toxin injections must be performed exclusively by trained medical professionals with appropriate anatomical knowledge. Early recognition of cosmetic injection–related strabismus is essential to avoid unnecessary neurological workup and to ensure timely conservative management.

## 1 Introduction

Botulinum toxin type A (BoNT‐A) is widely used for facial rejuvenation and is considered safe when administered by trained medical professionals [1, 2]. However, unintended toxin spread may result in extraocular muscle involvement—an underrecognized complication, particularly among non‐ophthalmic practitioners who frequently perform cosmetic injections [3, 4, 5, 6]. Although inferior and superior oblique dysfunctions are the most documented causes of diplopia after aesthetic injections [7, 8, 9], horizontal diplopia resulting from lateral rectus (LR) weakening is considerably less common, despite the lateral canthal region being one of the most commonly targeted sites in cosmetic practice [10, 11].

Recent dermatologic literature has highlighted this region's anatomical vulnerability. Khan et al. reported a case of LR palsy after crow's feet injections [10]. They emphasized that injections placed within 1–1.5 cm of the lateral orbital margin may predispose to toxin diffusion toward the LR muscle or its vascular supply [10]. As cosmetic BoNT‐A is increasingly administered in non‐medical settings, clinicians across dermatology, emergency care, and general medicine are increasingly encountering ocular complications that require ophthalmic evaluation [4, 5, 9]. Correctly identifying toxin‐induced ocular misalignment is essential to avoid unnecessary neuroimaging, prevent misdiagnosis, and ensure proper patient counseling [8, 10, 11].

**FIGURE 1:**
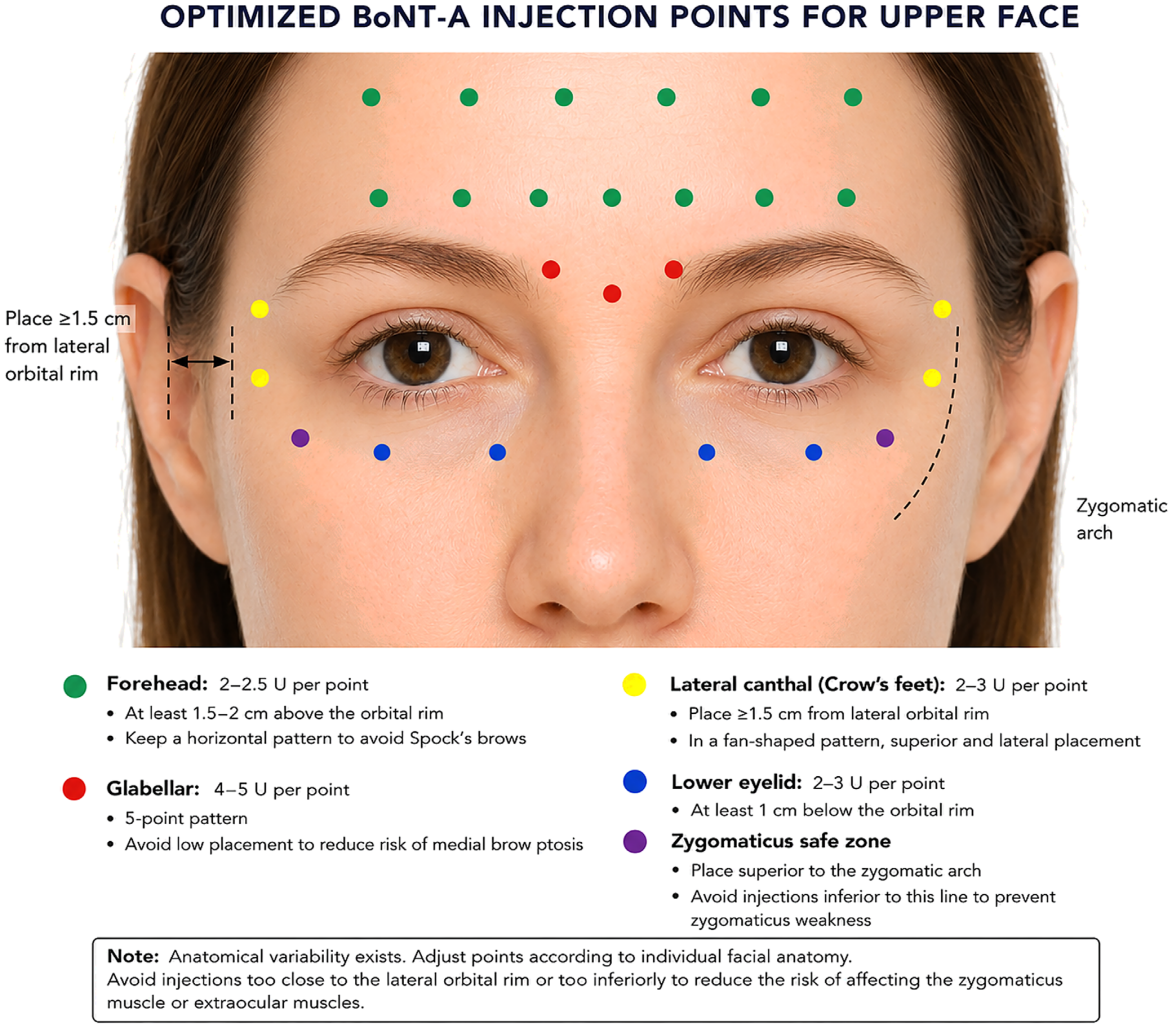
This diagram illustrates commonly used anatomical injection points for aesthetic botulinum toxin type A treatment of the upper face. Colored markers indicate recommended sites for glabellar complex relaxation (red), frontalis modulation (green), lateral canthal rhytids or “crow's feet” (purple), infraorbital smoothing (blue), and lateral border alignment (yellow). These points represent typical injection landmarks and are provided for reference; they do not depict the exact pattern used in the presented clinical case. Maintaining appropriate distance from the lateral orbital rim is essential to prevent toxin diffusion toward the lateral rectus muscle.

We present a case of acute esotropia following lateral canthal cosmetic BoNT‐A injection performed in a non‐medical beauty center. The clinical features were consistent with transient LR weakening. This case contributes to the growing recognition of LR involvement in periocular BoNT‐A complications and highlights the importance of anatomical awareness and interdisciplinary communication (Figure 1).

## 2 Case Report

A 39‐year‐old woman presented with a 1‐day history of painless horizontal binocular diplopia, particularly noticeable during distance fixation and computer use. Five days earlier, she had undergone cosmetic BoNT‐A injections to the glabellar, forehead, and bilateral lateral canthal regions at a non‐medical beauty center. The toxin brand, dilution, and dosage were unknown. Her medical and ocular histories were unremarkable.

**FIGURE 2:**
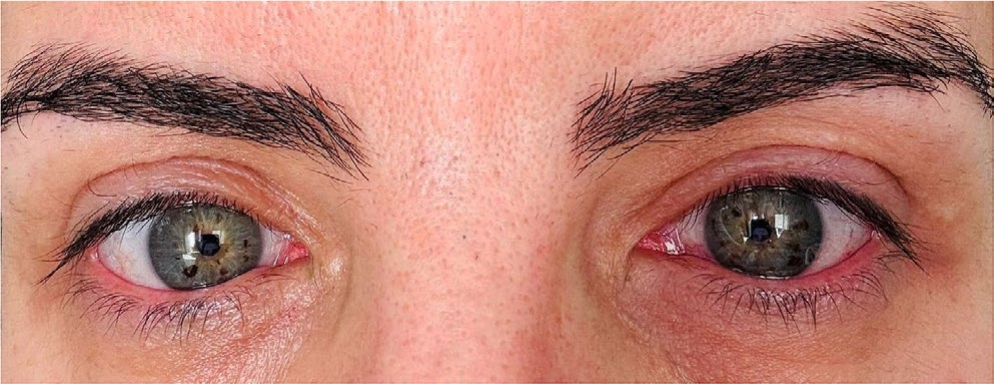
Frontal photograph demonstrating acute binocular misalignment in the patient following cosmetic periocular BoNT‐A administration. The right eye shows a subtle inward deviation relative to the left eye, consistent with small‐angle esotropia suggestive of lateral rectus weakening. Conjunctival injection and mild periorbital erythema are visible bilaterally. No ptosis is apparent. These findings correspond clinically with patient‐reported horizontal binocular diplopia and are compatible with toxin‐induced disruption of fusional mechanisms.

Best‐corrected visual acuity was 0.0 logMAR in both eyes. Pupils were normal, and slit‐lamp and fundus examinations were unremarkable. Cover testing revealed a comitant esotropia of approximately 10 prism‐diopters at both distance and near. Extraocular movements appeared full; however, subtle weakness of abduction could not be entirely excluded. Worth four‐dot testing demonstrated perception of five lights, consistent with uncrossed diplopia attributable to acute esotropia. Stereopsis was markedly reduced. Convergence amplitudes were normal, whereas divergence reserves were mildly diminished (Figure 2).

Neurological examination was regular. MRI of the brain and orbits showed no abnormalities. Given the temporal relationship to the injection and the binocular sensory findings, the clinical presentation was attributed to transient lateral rectus weakness due to unintended periocular diffusion of the toxin.

To alleviate symptoms, an 8‐prism‐diopter base‐out prism was prescribed—intentionally undercorrecting the measured 10‐PD deviation to support the patient's fusional capacity during early recovery. At 1 month, the deviation had decreased, allowing reduction of prism strength to 6 PD; at 2 months, the prism was reduced to 2 PD. By month 4, the patient was asymptomatic and no longer required prism correction. At the 6‐month follow‐up, ocular alignment and binocular function had fully normalized. This course was consistent with transient BoNT‐A–induced chemodenervation and expected neuromuscular recovery.

## 3 Discussion

Cosmetic botulinum toxin injections to the lateral canthal region are widely perceived as safe; however, this case demonstrates that even superficially administered injections can lead to unintended extraocular muscle involvement [11]. Transient esotropia secondary to lateral rectus weakening remains an under‐recognized complication, particularly among dermatology and aesthetic practitioners [5]. The subtle presentation in our patient—marked by preserved ductions despite clear sensory evidence of binocular disruption—broadens the known clinical spectrum of toxin‐induced LR dysfunction and highlights the diagnostic challenges that arise when motility deficits are mild or not overtly apparent [10, 11].

Khan et al. described a similar case of LR palsy following crow's feet injections, in which overt abduction limitation and small‐angle esotropia were attributed to inadvertent toxin diffusion into the LR muscle [10]. Their report emphasized that injections placed within 1–1.5 cm of the lateral orbital rim may increase the risk of spreading toward the muscle or its vascular supply [10, 12]. In contrast to their patient, our case demonstrated no measurable motility deficit, despite showing reduced divergence, impaired stereopsis, and a five‐light Worth pattern—all strongly suggestive of subtle LR weakening. This comparison highlights that toxin‐induced LR involvement exists on a continuum ranging from overt paresis to more nuanced sensory‐driven fusional breakdown. Recognizing this spectrum is crucial to avoid misclassification as functional or neurological disorders, primarily when injections are performed in non‐medical settings or by providers with limited anatomical training [4, 5].

The onset of symptoms 5 days after injection and complete resolution by 6 months mirrors the known pharmacodynamics of BoNT‐A [13]. The recovery timeline in our patient also aligns with the four‐month improvement reported by Khan et al. [10], reinforcing the reversible and self‐limiting nature of toxin‐induced ocular misalignment. Conservative management remains appropriate, and temporary prism correction—including mild undercorrection to support fusional mechanisms—can provide effective symptomatic relief during the recovery phase [11].

Anatomically, the lateral canthal region contains vascular pathways adjacent to the LR muscle, providing potential routes for toxin to diffuse even when injections appear superficially placed [10, 12]. This reinforces the importance of avoiding deep penetration and maintaining a safe distance from the lateral orbital margin. Greater awareness of these anatomical considerations may reduce the risk of inadvertent exposure [10, 12].

Finally, this case emphasizes the broader public health issue surrounding unregulated cosmetic procedures. Patients often present first to dermatologists or general practitioners, and early recognition of BoNT‐A–related diplopia can prevent unnecessary investigations and inappropriate concern [4, 9]. Awareness that toxin‐induced diplopia is typically reversible allows clinicians to provide accurate reassurance and timely referral [11, 13].

## 4 Conclusion

Lateral rectus weakening is a rare but clinically meaningful complication of cosmetic BoNT‐A injections in the lateral canthal region. Even when ocular motility appears intact, subtle sensory disturbances may reveal early LR dysfunction induced by the toxin. The condition is typically benign, self‐limiting, and effectively managed with conservative measures such as temporary prism correction. Increased awareness among ophthalmologists, dermatologists, and aesthetic practitioners is crucial to ensure early recognition, appropriate counseling, and safe cosmetic practice. This case highlights the necessity for anatomical expertise in cosmetic BoNT‐A administration and underscores the risks associated with injections performed in non‐medical environments.

## Author Contributions

Tunahan Akyol: Conceptualization, clinical examination, data interpretation, manuscript drafting, critical revision, and final approval. Emine Seker Un: Data collection, literature review, manuscript editing. Osman Parca: Manuscript revision, figure preparation, and supervision. All authors have read and approved the final manuscript and meet the ICMJE criteria for authorship.

## Funding

The authors have nothing to report.

## Ethics Statement

This case report was conducted in accordance with the principles of the Declaration of Helsinki. Ethical approval was not required for single‐patient case reports according to the Pamukkale University Non‐Interventional Clinical Research Ethics Committee.

## Consent

Written informed consent was obtained from the patient for publication of the details of her medical case and accompanying images.

## Conflicts of Interest

The authors declare no conflicts of interest.

## Data Availability

All data supporting the findings of this case report are included within the manuscript. Additional clinical information is available from the corresponding author upon reasonable request. No publicly available datasets were generated or analyzed for this study.
